# Inner limiting membrane bridges within Bruch’s membrane defects in pathological myopia

**DOI:** 10.1038/s41598-022-26075-4

**Published:** 2022-12-10

**Authors:** Songhomitra Panda-Jonas, Jost B. Jonas, Rahul A. Jonas

**Affiliations:** 1 Department of Ophthalmology, University of Heidelberg, 69120 Heidelberg, Germany; 2 Privatpraxis Prof Jonas und Dr Panda-Jonas, Heidelberg, Germany; 3 Department of Ophthalmology, Medical Faculty Mannheim, Heidelberg University, Universitäts-Augenklinik, Theodor-Kutzer-Ufer 1-3, 68167 Mannheim, Germany; 4 Institute of Molecular and Clinical Ophthalmology, Basel, Switzerland; 5 Department of Ophthalmology, University Hospital of Cologne, Cologne, Germany

**Keywords:** Refractive errors, Retinal diseases

## Abstract

The purpose of the study was to examine peculiarities of the inner limiting membrane (ILM) in axially elongated eyes. The histomorphometric study included human globes enucleated due to reasons such as painful secondary angle-closure glaucoma or malignant uveal melanomas. Using light microscopy, we searched for regions with ILM-specific features in association with a marked axial elongation. Out of 279 eyes (279 patients) (mean age: 61.8 ± 13.9 years; axial length: 25.5 ± 3.1 mm (range: 20.0–37.0 mm)), two eyes (axial length: 30 mm and 34 mm, respectively) showed one region and two regions, respectively, characterized by ILM presence and absence of all other retinal layers, retinal pigment epithelium, Bruch´s membrane (BM) and choroid. The length of these regions, called ILM-bridges, was 1.06 mm, 0.73 mm, and 0.62 mm, respectively. All ILM-bridges were spatially associated with a larger, underlying BM defect and with localized scleral thinning without a staphylomatous scleral configuration. The distance between the ILM-bridges and the optic disc ranged between 1.92 mm and 4.21 mm. In univariable analysis, ILM-bridge number increased with longer axial length (beta: 0.19; *P* = 0.002) and higher BM defect prevalence (beta: 0.21; *P* = 0.001), while in multivariable analysis, the ILM-bridges number remained to be significantly correlated only with a higher prevalence of BM defect (beta: 0.15; *P* = 0.048). ILM-bridges occur in eyes with pathologic myopia in spatial association with underlying, larger BM defects. They may be due to an axial elongation-associated local stretching and rupture of all other retinal layers, caused by the BM defect-related enlargement of the retinal undersurface. Future studies may explore whether these histologic observations support the notion of the ILM having a relatively high biomechanical strength against myopic stretching-associated forces.

## Introduction

Myopic axial elongation is associated with an increase in the ocular diameters, mostly in the sagittal direction, and to a markedly lower degree in the coronal plane^1–3^. With the myopic enlargement of the globe wall occurring predominantly in the equatorial and retro-equatorial region, myopic axial elongation leads to an increased distance between the ora serrata and the optic disc^4,5^. Since the inner limiting membrane (ILM) and the layer of the retinal nerve fibers as the axons of the retinal ganglion cells form the only area-wide connection between all other retinal layers and the optic disc, any increase in the ora serrata-optic disc distance leads to an elongation, and potentially stretching, of the ILM and retinal nerve fibers. The ILM is the basal membrane of the Müller glial cells, the cell bodies of which are located in the retinal inner nuclear layer and which extend from the external limiting membrane as a converging structure of the Müller cell foot plates to the ILM. As basal membrane of the Müller cells, the ILM may have similarities with the lens capsule as the basal membrane of the lens epithelium cells and with the basal membrane of the retinal pigment epithelium (RPE) lying on top of, or forming part of, Bruch’s membrane (BM). The biomechanical properties of the ILM have not been examined under standardized conditions yet, so that it has been unknown how the ILM reacts to its axial elongation-associated lengthening and potential stretching. Here we histomorphometrically examined highly myopic eyes and searched for ILM-specific features in association with a marked axial elongation.

## Methods

The histomorphometric study included human globes which had been enucleated due to reasons such as malignant uveal melanomas or painful secondary angle-closure glaucoma. According to the guidelines laid down in the World Medical Association Declaration of Helsinki, the Medical Ethics Committee II of the Medical Faculty Mannheim of the Heidelberg University approved the study and waived the necessity of an informed written consent signed by the study participants, since the enucleation had been performed about 25–60 years before the investigation was performed. The globes had already been included in previous studies addressing different topics^6,7^.

Immediately after enucleation, the globes had been fixed in a solution of 4% formaldehyde and 1% glutaraldehyde for one week at room temperature. Before fixation, the globes had not been cut open nor had the preservative agent been injected intravitreally. As also described in detail previously, we measured the sagittal, horizontal and vertical globe diameters^6,7^. Out of the fixed globes, we removed the middle part with a thickness of approximately 8 mm. It included the optic nerve head, the pupil and the macular region, except for eyes with a malignant melanoma, in the case of which the middle segment ran in an anterior–posterior direction through the tumor and the pupil and optic nerve head. We dehydrated the middle segment in alcohol, imbedded it in paraffin and sectioned it for light microscopy. We used the Periodic-Acid-Schiff method or hematoxylin–eosin to stain the tissue. For further evaluation, we took one section with a thickness of approximately 5–8 µm and which ran through the central part of the pupil and optic nerve head.

Using light microscopy, we examined the slides of all eyes and searched for regions characterized by the presence of ILM and absence of most other retinal layers. These regions were defined as “ILM-bridges”. Using a millimeter scale built into the objective of the microscope, we measured the thickness of the anatomical structures, including the length of the ILM-bridges, and distances to landmarks, such as the optic nerve head. We defined high myopic axial elongation by an axial length of ≥ 26.0 mm.

Using a statistical software program (SPSS for Windows, version 27.0; IBM-SPSS, Chicago, Illinois, USA), we assessed the mean values, standard deviations and 95% confidence intervals (CI) of the measured parameters. Applying regression analyses, we tested associations between these parameters and other histomorphometric parameters such as axial length. We calculated the standardized regression coefficient beta and the non-standardized regression coefficient B and its 95% CI in linear regression analyses, and we assessed the odds ratios (ORs) and their 95% CI in binary regression analysis. The level of significance was 0.05 (two-sided) in all statistical tests.

## Results

The study included 279 eyes (279 patients; 151 (54.1%) men) with a mean age of 61.8 ± 13.9 years (median: 63.0 years; range: 24–89 years) and a mean axial length of 25.5 ± 3.1 mm (median: 24.0 mm; range: 20.0–37.0 mm). Out of the 279 eyes, 95 (34.1%) eyes had an axial length of ≥ 26.0 mm, and 47 (16.8%) had an axial length of ≥ 29.0 mm.

**Figure 1:**
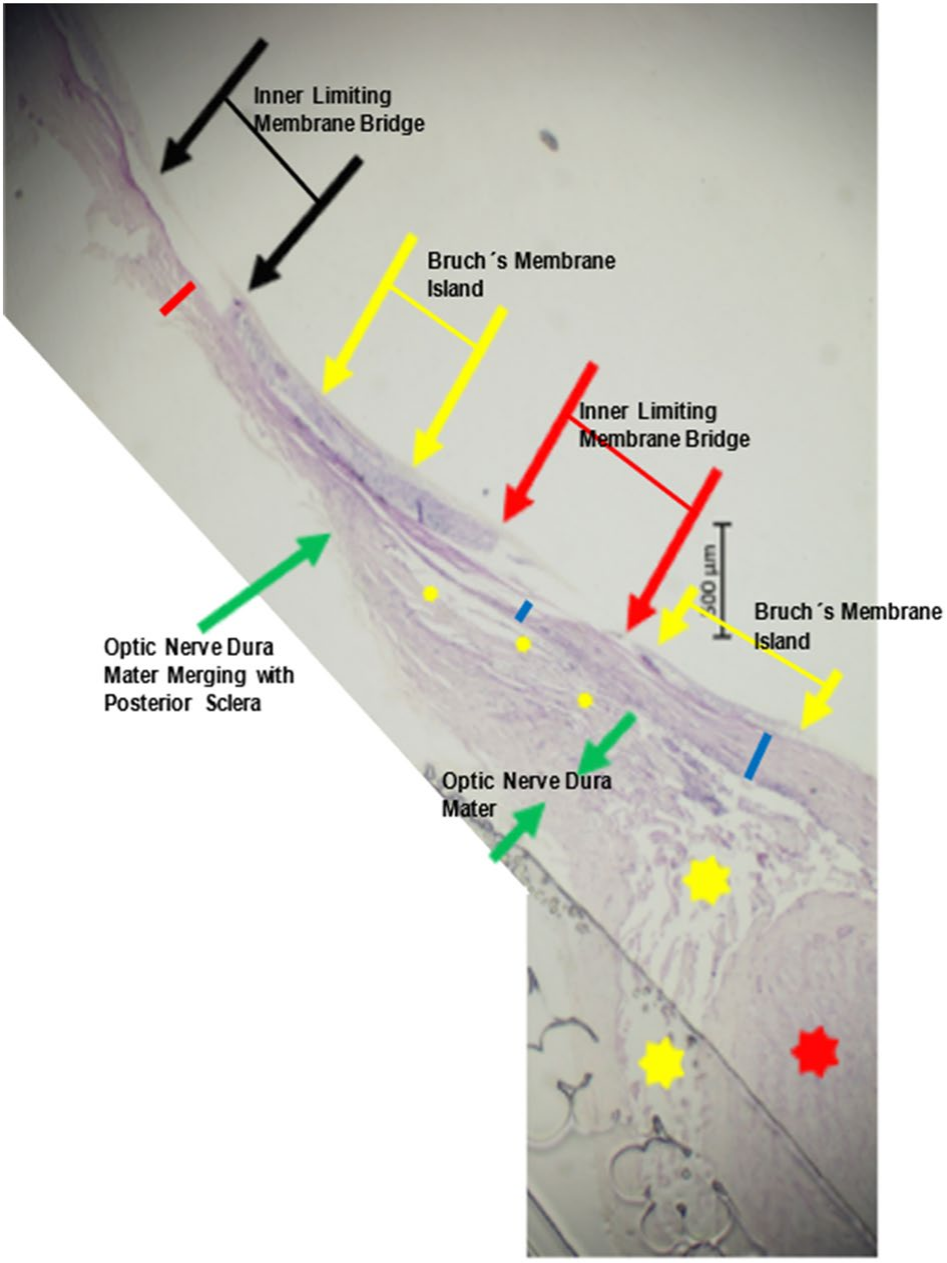
Histo-photograph showing the retina of an extremely elongated eye (axial length: 30.0 mm) with two regions (“inner limiting membrane (ILM) bridge”) (between both black arrows (length: 0.62 mm) and between both red arrows (length: 0.73 mm)) in which the retina consisted only of the inner limiting membrane; long green arrow; merging point of optic nerve dura mater (between both short green arrows) with the posterior sclera; two blue bars: peripapillary scleral flange (thickness: 230 µm and 80 µm), ending at the merging point of the optic nerve dura mater with the posterior sclera; yellow (small and large) asterisks: orbital cerebrospinal fluid space; red asterisks: optic nerve; red bar: scleral thickness of 173 µm; distance between the right ILM-bridge and the optic disc border: 1.92 mm; distance between both ILM-bridges: 1.56 mm; length of ILM-bridges: 0.73 mm (right ILM-bridge) and 0.62 mm (left ILM-bridge).

**Figure 2:**
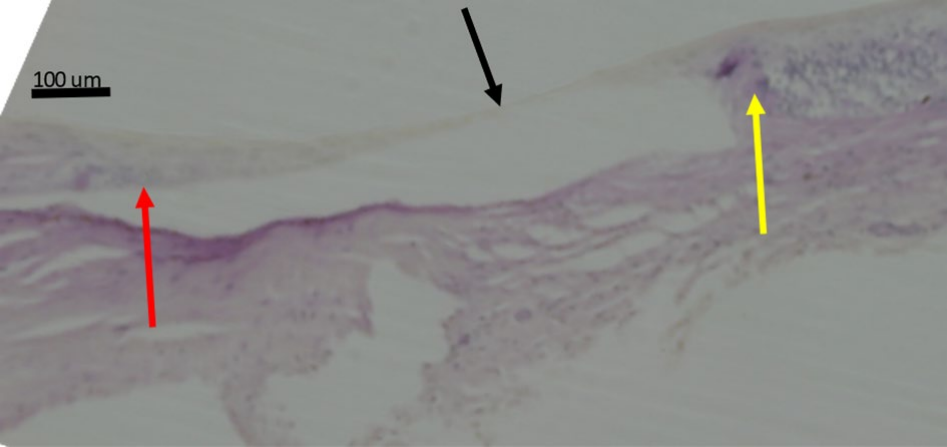
Histo-photograph showing an inner limiting membrane (ILM) defect (please see Fig. 1) in greater magnification (black arrow), yellow and red arrow: ends of the retinal layers (except for the ILM) ending abruptly (yellow arrow) or smoothly (red arrow); in the ILM-defect region, the retina consists only of the inner limiting membrane, with the retinal pigment epithelium, Bruch’s membrane and choroidal structures additionally absent.

**Figure 3:**
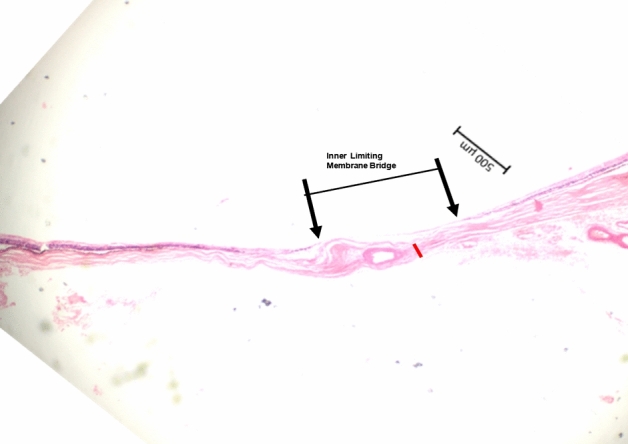
Histo-photograph showing the retina of an extremely elongated eye (axial length: 34.0 mm) with a region (between both black arrows) in which the retina consisted only of the inner limiting membrane (ILM) (“ILM-bridge”); length of the ILM-bridge: 1.04 mm; scleral thickness 110 µm (red bar): distance to the optic disc border: 3.72 mm.

**Figure 4:**
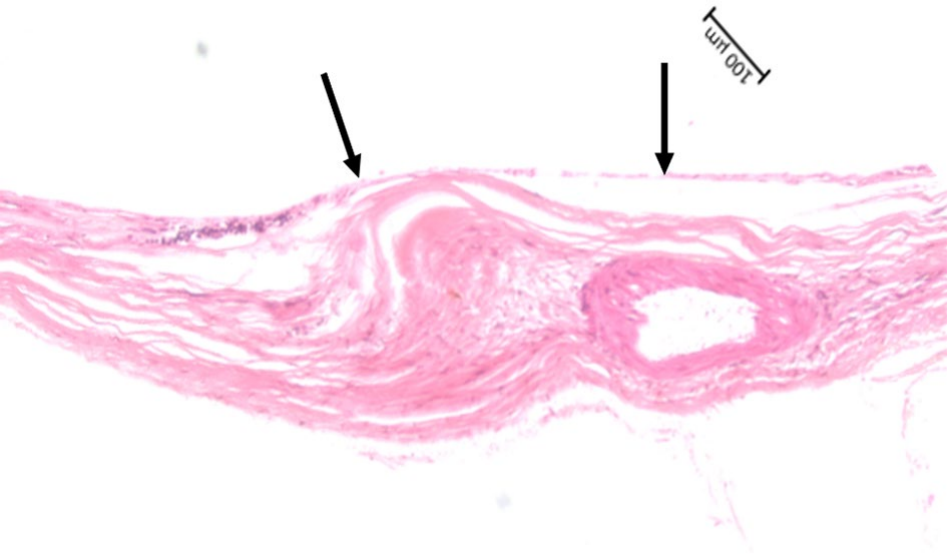
Histo-photograph showing the inner limiting membrane bridge in greater magnification (between black arrows), with few cells attached to the inner limiting membrane.

The slides of two eyes of two patients (1 man), aged 63 years and 65 years and with an axial length of 30 mm and 34 mm, respectively, showed one region and two regions, respectively, which were characterized by the presence of the ILM, while all other layers of the retina and the RPE, BM and choroid were absent (Figs. 1, 2, 3, 4). In the eye with two ILM-bridges (axial length: 30.0 mm), the distance between the optic disc border (defined as the end of the lamina cribrosa) and the ILM-bridge located closest to the optic disc was 1.92 mm, the length of the ILM-bridge was 0.73 mm, and the length of tissue between both ILM-bridges was 1.56 mm, so that the distance between the disc border and the second ILM-bridge was 4.21 mm (Figs. 1, 2). The length of the second ILM-bridge was 0.62 mm, with an underlying BM defect with a length of 1.30 mm. The region between the two ILM-bridges showed a BM island with a localized proliferation of RPE cells. In direction to the optic nerve head, the ILM-bridge (located next to the optic nerve head) bordered a parapapillary region with a remnant of BM (length: 0.77 mm), at the end of which parapapillary gamma zone with a length of 0.99 mm started in direction to the optic disc border.

In the eye with one ILM-bridge, the distance between the ILM-bridge and the optic disc border was 3.72 mm, and the length of the ILM-bridge was 1.06 mm (Figs. 3, 4). The ILM-bridge was spatially associated with a BM defect of a length of 1.88 mm, and the scleral thickness measured 110 µm. All ILM-bridge regions were characterized by the absence of all retinal layers (except for the ILM), of the RPE, BM and of all choroidal layers (Figs. 1, 2, 3, 4). The sclera was thinner in the region of the ILM-bridges than in the collateral areas. All three ILM-bridges were located relatively close to the optic nerve head. None of these regions showed a staphylomatous configuration of the sclera.

**Figure 5:**
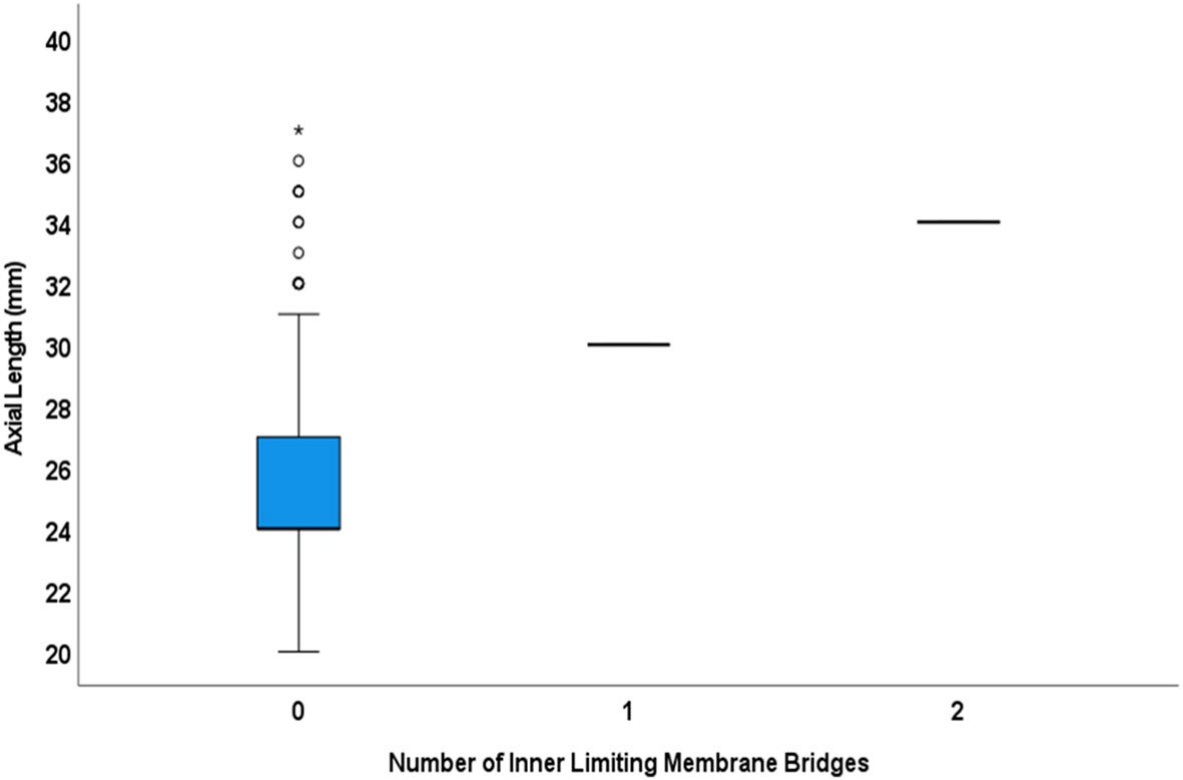
Boxplot showing the distribution of the number of inner limiting membrane bridges and axial length.

**Figure 6:**
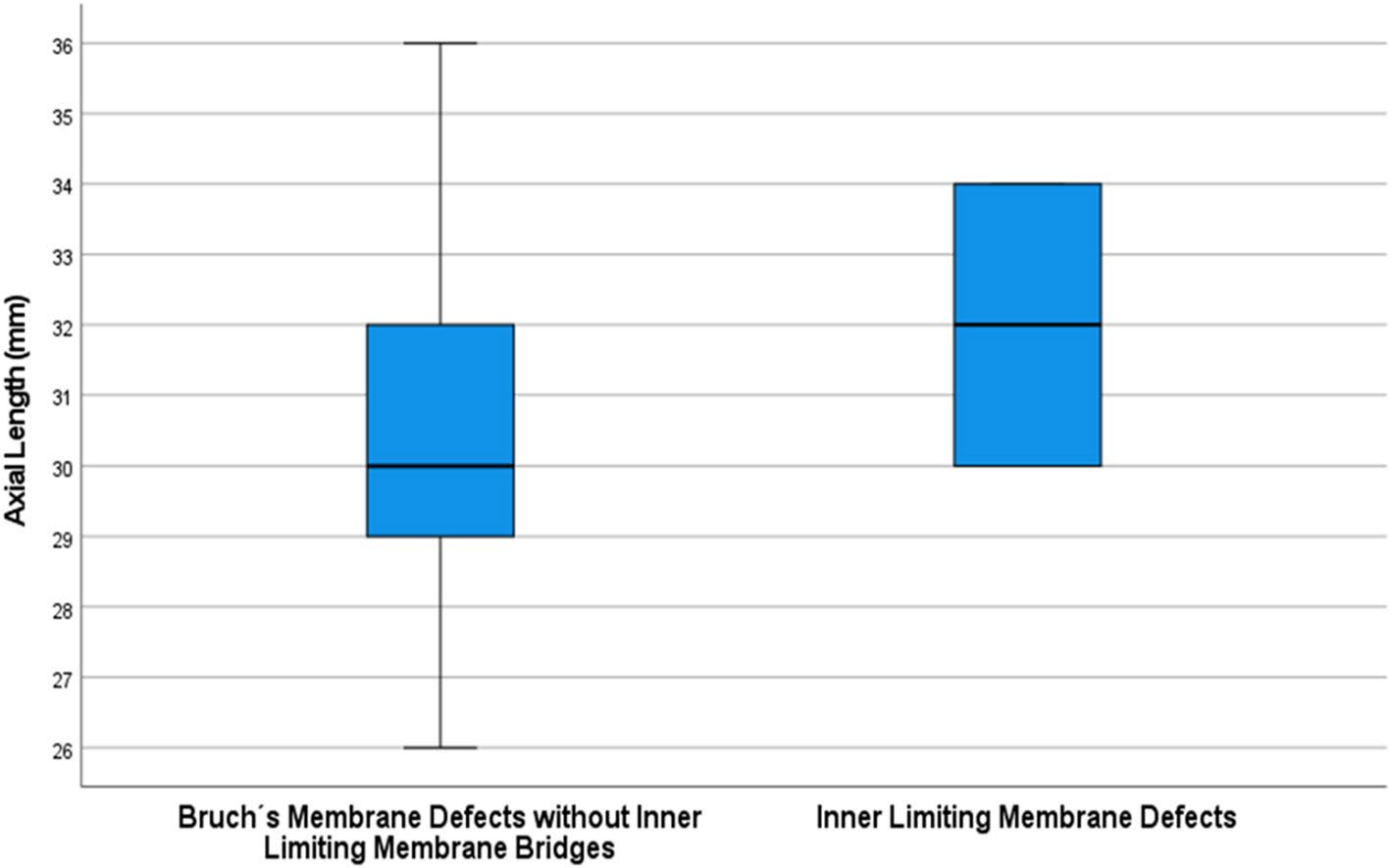
Boxplot showing the distribution of axial length stratified by the presence of inner limiting membrane (ILM) bridges (with Bruch’s membrane defects) versus the presence of Bruch’s membrane defects without ILM-bridges.

In univariable analysis, the number of the ILM-bridges increased significantly with longer axial length (beta: 0.19; B: 0.08; 95% CI 0003, 0.013; *P* = 0.002) and higher prevalence of BM defects (beta: 0.21; B: 0.10; 95% CI 0.04, 0.15; *P* = 0.001) (Fig. 5). In a multivariable analysis with axial length and prevalence of BM defects as independent variables, the number of ILM-bridges remained to be significantly correlated only with a higher prevalence of BM defects (beta: 0.15; B: 0.07; 95% CI 0.001, 0.14; *P* = 0.048). Eyes with ILM-bridges as compared to eyes with BM defects without associated ILM-bridges had a longer, but not significantly (*P* = 0.32) longer, axial length (32.0 ± 2.8 mm versus 30.3 ± 2.4 mm) (Fig. 6). The prevalence of BM defects was associated with longer axial length (odds ratio: 1.71; 95% CI 1.43, 2.04; *P* < 0.001).

## Discussion

This histomorphometric study revealed that extremely elongated eyes can show areas in which, except for the ILM and a locally thinned sclera, all other tissues including all other retinal layers, the RPE, BM, and the whole choroid were absent. These areas, called “ILM-bridges”, were not detected in eyes with an axial length of less than 30 mm. The ILM-bridges were smaller than the underlying BM defects. The number of the ILM-bridges increased significantly with a higher prevalence of BM defects. The distance between ILM-bridges and the optic disc border ranged between 1.92 and 4.21 mm. Although spatially associated with a localized thinning of the underlying sclera, the latter did not show a staphylomatous configuration.

Since ILM-bridges have not been described before, findings of the study cannot directly be compared with observations made in previous investigations^8^. The occurrence of ILM-bridges in extremely elongated eyes belong to a panoply of features of highly myopic eyes, such as an elongation and thinning of the peripapillary scleral flange, peripapillary choroidal border tissue and lamina cribrosa; a marked thinning of the choroid, most markedly in the posterior region and most pronounced for the choroidal layers with the middle-sized and large choroidal vessels; a thinning of the sclera, most marked at the posterior pole; widening of the BM opening of the optic nerve head and enlargement of the optic nerve head canal; and development of macular BM defects, to mention only a few^1–3,8^. ILM-bridges were found only in areas with underlying BM defects which, as compared with ILM-bridges, were wider. In addition, eyes with ILM-bridges (and with BM defects) as compared to eyes with BM defects without ILM-bridges had a longer, but not significantly longer, axial length. One may infer that the axial elongation may have led first to the development of BM defects which eventually led to the formation of ILM-bridges.

The reasons for an etiologic association between BM defects and ILM bridges as well as for the development of BM defects have remained elusive so far. It has recently been discussed that myopic axial elongation occurs in association with an enlargement of BM mainly in the retro-equatorial and equatorial region, pushing the posterior BM backward with the sequel of a compression and thinning of the posterior choroid^9^. In such a model, the axial-elongation associated thinning of the sclera would occur in a secondary manner. Supporting the notion of BM instead of the sclera as the driver of axial elongation is that a primary enlargement and elongation of the sclera would widen the choroidal space. In addition, the thickness of BM, in contrast to the thickness of the sclera, did not decrease with longer axial length^10^. The BM enlargement taking place in particular in the retro-equatorial region may be associated with the observed enlargement of the coronary globe diameters and may lead, through an increased strain within BM, primarily to an enlargement of the BM opening of the optic nerve head^10,11^. In a second step, additional BM defects may develop in the macular region. These macular BM defects lead to a localized lengthening and stretching of the overlying retina. Correspondingly, areas with BM defects usually are free of retinal photoreceptors and are usually covered only by the middle and inner retinal layers^7^. In the case of ILM-bridges, the localized stretching of the retinal layers, caused by the enlargement of the retinal undersurface in the area of the BM defect, might have caused an enlarging defect within the retina with a retraction of the middle and inner retinal layers, leaving the ILM as the only remaining retinal structure covering the BM defect.

Since the ILM has remained the only retinal structure connecting the retinal regions peripheral to the ILM-bridge with the optic disc, one may discuss that the biomechanical strength, including the resistance against the retinal elongation-induced force, might perhaps be stronger in the ILM than in the other retinal layers. It refers in particular to the layer of the retinal nerve fibers which, besides the ILM, is the only other structure with an area-wide connection with the optic disc. The notion of a relatively high biomechanical strength of the ILM can only be substantiated by biomechanical tests measuring the plasticity and elasticity of the separated ILM in dependence of the force experimentally exerted on it. Interestingly, the ILM-bridges were located in regions with a localized scleral thinning, however without a staphylomatous configuration. Previous studies have shown an association between scleral staphylomas and myopic maculoschisis in highly myopic eyes^12^. One may discuss that if the sclera in these eyes shows an outpouching in the form a staphyloma, the ILM firmly connected with the optic disc cannot follow the increased circumference in the area of the staphyloma. In contrast, BM can slide away from the optic disc border and remain in contact with the choroid and elongated sclera. The sliding of BM is possible due to an elongation and thinning of the peripapillary choroidal border tissue, which connects BM end with the interface between the lamina cribrosa and the peripapillary scleral flange^13^. Simultaneously, the elongation and thinning of the peripapillary choroidal border tissue leads to the development of parapapillary gamma zone^3,7^. The situation with the ILM tautly connected strung to the optic disc and BM slipping away, may lead to an intraretinal strain in sagittal direction. It may secondarily result in a partial separation between the inner retinal layers, i.e., mainly the ILM in connection with the retinal nerve finer layer, and the outer retinal layers, which, through the retinal photoreceptors and the RPE, are relatively firmly connected with the BM. Such an intraretinal, horizontal and incomplete cleavage may lead to a myopic maculoschisis. Such a notion is supported by the observations made in the present study suggesting that the ILM has the highest biomechanical strength of all retinal layers.

If one assumes that the absence of retinal nerve fibers in the region of the ILM-bridges was not due to a glaucomatous process, one may discuss that the local tissue stretching within the BM defect area might have disrupted not only the deep and middle retinal layers but also the inner retinal layer, i.e., the retinal nerve fiber layer. Such a notion fits with the occurrence of a non-glaucomatous optic nerve damage in highly myopic eyes, as also described in the recent Ural Eye and Medical Study^14^. The local tissue stretching in the area of the BM defect may add to the axial elongation-associated lengthening and potentially stretching of the retinal nerve fibers on their way from the peripheral retinal ganglion cells to the optic disc. In particular, the development of a temporal parapapillary gamma zone leads to an increase in the disc-fovea distance including a lengthening of the papillomacular retinal nerve fibers^15^. From a perimetric point of view, the occurrence of ILM-bridges in the area of a BM defect adds an arcuate visual defect, caused by the loss of the retinal nerve fibers in that area, to an areolar perimetric defect, caused by the absence of photoreceptors in the region of the BM defect^7^.

The limitations of our study should be considered: First, serial sections of the globes were not available, so that the number of ILM-bridges per eye might have been higher than we detected. Second, the patients whose eyes were included into the study were not recruited in a population-based manner, so that the study could not provide data on the prevalence of ILM-bridges in the general population. Third, the study material of our investigation might have had a selection bias since it was based on human globes enucleated for clinical reasons. The findings of our study may therefore not directly be transferred to eyes without these diseases. Fourth, tissue swelling due to the ischemia occurring after enucleation and before fixation and fixation-related shrinkage of the tissue are unavoidable post enucleation changes which may have influenced the dimensions of the ocular tissues in our study. Also, mechanically induced changes during the histological processing of the globes may have altered the tissues. Fifth, the patients included in our study were predominantly of European descent. Future studies may address whether the findings obtained in our investigation can be used to draw an inference about individuals of other ethnicities.

In conclusion, ILM-bridges occur in eyes with pathologic myopia and are found in spatial association with underlying, larger BM defects. ILM-bridges may be due to a local stretching and rupture of all other retinal layers, caused by the BM defect-related enlargement of the retinal undersurface. Future investigations may address whether these histologic findings support the notion of the ILM having a relatively high biomechanical strength to resist a myopic stretching-associated force.

## Data Availability

The datasets used and/or analyzed during the current study are available from the corresponding author on reasonable request. Financial Disclosures: All authors: European patent application 16 720 043.5 and US patent application US 2019 0085065 A1: Agents for use in the therapeutic or prophylactic treatment of myopia or hyperopia).
